# The meaning of the name of ‘pulmonary rehabilitation’ and its influence on engagement with individuals with chronic lung disease

**DOI:** 10.1177/1479973119847659

**Published:** 2019-05-28

**Authors:** Rebecca Oxley, Samantha L. Harrison, Arthur Rose, Jane Macnaughton

**Affiliations:** 1 The Centre for Medical Humanities, Caedmon Building, Durham University, Durham, UK; 2 School of Health and Social Care, Centuria Building, Teesside University, Middlesbrough, Tees Valley, UK

**Keywords:** Pulmonary rehabilitation, breathlessness, medical humanities, singing for lung health, dance

## Abstract

Pulmonary rehabilitation (PR) is recommended for all individuals living with a lung condition and chronic breathlessness. This article considers how adopting an interdisciplinary, medical humanities approach to the term ‘pulmonary rehabilitation’ might unpack some of the misconceptions, misrepresentations or negative connotations surrounding it, which have been largely overlooked in explanations of the low uptake of this programme. Taking key insights from Wellcome Trust-funded *Life of Breath* project, including ethnographic research in community fitness groups in North East England and the ‘Breath Lab’ special interest group, this article outlines how the whole-body approach of PR is not easily understood by those with lung conditions; how experience can inform breath perception through the pacing of everyday life; and how stigma can impact rehabilitation. This article highlights the value of medical humanities in working through communicative challenges evident in the translation of PR between patient and clinical contexts and sets out two arts-based approaches (Singing for Lung Health and dance movement) as potential options that could be included in the PR referral. Finally, the article outlines the need for collaborative research exploring the communication and meaning of healthcare strategies and experiences at the interface of the arts, humanities and medical practice.

## Introduction

Pulmonary rehabilitation (PR), comprising an endurance-based exercise programme and condition-specific education, is recommended for all individuals living with a lung condition and chronic breathlessness.^1^ Comprehensive PR programmes run over a minimum of 6 weeks, with at least two sessions per week supervised by a multidisciplinary team.^1^ Programmes are available in various locations including: hospital inpatient, hospital outpatient, the community and in the home.^1,2^ There are continuing challenges regarding uptake and engagement in PR services. Despite its recognised benefits in improving quality of life, enhancing functional exercise capacity, reducing symptoms of anxiety and depression and preventing hospital readmissions in those who attend and complete, approximately 20–60% of those eligible do not complete programmes.^3,4^ The number of individuals able to complete programmes is further diminished (9%) in those who have recently suffered an acute exacerbation (defined as an increase in symptoms and often resulting in an admission to hospital).^5,6^

While sociodemographic data and clinical variables appear to explain some variance in completion rates, psychological variables have shown more promise. Heightened experiences of depression have led to increased drop-out rates in those whose disease-symptoms remained stable. Notably higher levels of anxiety were observed in those who suffered an acute exacerbation and discontinued PR compared to those who completed the programme.^6,7^ That said, reasons for poor PR uptake and engagement are likely very complex and informed by sociocultural factors such as education level, religious beliefs and ethnicity. Recent work has looked at participation in PR noting that key issues include referral factors, such as lack of understanding what to expect; the importance of patient beliefs, such as fears about the effects of exercise on breathlessness or other symptoms; and social factors such as the support of family and friends, and adjustment to the programme, but the influence of language on understanding or expectations was not mentioned.^3,8,9^

The ‘evidence gap’ cited in clinical research has focused on how patients’ physical and cognitive abilities relate to their ability or inclination to access PR services, rather than analysing how the ways that those services are presented to patients may affect their emotional connection with them.^10^ While emerging qualitative research with individuals following an acute exacerbation of Chronic Obstructive Pulmonary Disease (COPD) discusses the role of self-conscious emotions (i.e. shame, guilt) in patients declining referral to PR,^11^ connotations of the term ‘pulmonary rehabilitation’ itself has been largely overlooked, and the implications this might have for service provision have not yet been considered.

This article considers how adopting an interdisciplinary, medical humanities approach to the term might unpack some of the misconceptions, misrepresentations or negative connotations surrounding ‘pulmonary rehabilitation’. In order to understand these issues in greater depth, the term is approached from a variety of perspectives outside those typically included in respiratory research. So, in addition to a PR specialist, the authors included an anthropologist to attend to the lived experience of those who have attended PR (A1), a literary scholar to advise on the semantic associations of negative language, and a medical humanities researcher to negotiate these mixed methodologies. This perspective has been developed through drawing upon a range of research activities from the interdisciplinary Wellcome Trust-funded *Life of Breath* project.^12^ These research activities have highlighted that the significance of breath, the lungs and the body is contextual, and we need to pay attention to knowledge about how lives are lived outside the clinical context, and understand the factors that influence how people’s histories, culture and imagination influence their experience.^13–16^

This article is based upon work undertaken in the North East of England, a region of the United Kingdom where COPD is more prevalent than for the rest of the United Kingdom, and where 4 of the 10 so-called ‘hot spots’ for COPD are located, reflecting the higher levels of social deprivation of this area.^17,18^ The demographic of this region is largely white and the population with whom we interacted are mainly from lower socio-economic areas and in older age groups (aged over 50 years). Our article is contextualised to this demographic potentially limiting the generalisability of the insights drawn and does not report on cross-cultural insights into the nuances of language about breath. However, our work on *Life of Breath* work asserts the importance of culturally nuanced research especially about language^19^ and demonstrates the power language can have on the decisions patients may make about their health. The insights presented in this article are likely to be useful to clinicians working with breathless patients in PR and also wider groups interested in encouraging patients to sustain physical activity following formal PR.

This article will, first, outline the range of ways in which the language of PR has the potential to obstruct uptake or engagement with this important approach to management; and, second, we will propose two new arts-based adjunctive approaches to breathlessness management which may be useful in helping patients live with breathlessness in ways that connect with their day to day lives and contexts.

## Methods

The *Life of Breath* project explores how people live with breathlessness by investigating experience alongside understanding of the historical, cultural, philosophical and spiritual ideas that may underpin that experience. The project also examines the influence of language, imagination and feelings on how and whether people feel able to undertake activity. This article draws upon information gleaned from a number of activities conducted as part of the *Life of Breath* project, including literature and narrative studies, and empirical, ethnographic research, namely participant observation and semi-structured interviews with those involved in community fitness groups for those living with breathlessness in North East England (conducted from 2015 to 2017).

One important interdisciplinary activity for the *Life of Breath* project is what we called ‘Breath Lab’. Breath Lab is a special interest group designed to bring together people from various clinical and non-clinical backgrounds, including patient representatives to think through issues pertinent to breath, breathing and breathlessness in a non-clinical environment. Empirical work undertaken on the project has revealed that patients and healthcare providers are often at odds in the words they use to express, define and approach breathlessness and its care, which can impact early intervention, diagnosis and treatment.^13^ This disconnect may be understood as a failure to translate, here understood as an attempt to convey a term meaningfully across different linguistic, cultural or social contexts.

## Findings

### Lung-centred rehabilitation?

During our Breath Lab PR emerged as a particularly difficult term to translate across contexts. The most pressing issue for those living with a lung condition who might not identify as an ‘expert patient’ was what PR was and what participation might involve. Those with chronic breathlessness suggested the term was ‘arcane’, ‘difficult to connect with’ and too highly medicalised to be understood. While this might imply the need for greater health literacy – teaching patients what PR means, a more nuanced approach may be required if we are to unpick how PR is perceived. What the term PR promotes and what it provides can have very different meanings. For example, the word ‘pulmonary’ is defined as ‘relating to the lungs’ which implies that PR would centre on this organ, when in fact the programme promotes whole body aerobic fitness and aims to provide the means for effective self-management. Furthermore, the proportion of individuals attending PR who have at least one other co-existing condition is between 51% and 96% meaning that motivation to engage in therapy ‘relating to the lungs’ may be overcome by the desire to attend to conditions not directly related to their respiratory disease.^7,11^

Our inquiries through ‘Breath Lab’ and ethnographic research conducted through the *Life of Breath* project have indicated that there is a hesitation to pursue PR due to a lack of clarity as to what the programme involves and what this means for individuals. This empirical research has revealed that many of those consulted with a lung condition initially considered that PR comprised breathing techniques, while others were concerned with the probability of compulsory, intense exercise. Certain individuals also reported that they attended community fitness groups for those with breathlessness ahead of PR to familiarise themselves with exercise, which runs contrary to referral process practice. Some PR-providers have offered a ‘taster’ session pre-PR or a familiarisation trial for the 6-min walk test. Opt-in sessions have shown some benefits on the uptake and retention of PR.^20^ In each case, however, we observed a mismatch between clinician and patient understanding of even the most basic words used to describe elements of the programme, such as ‘gentle’ exercise, sometimes leading to distressful experiences of rehabilitation. While most of those patients consulted, patient support groups and charity campaigns have sought to address uncertainty about the content and intensity of PR and to limit anxiety about performance from peers and healthcare professionals.

### Rehabilitation and perceptions of breathlessness

The term ‘rehabilitation’ fits more closely than ‘pulmonary’ with what PR provides, although, as with most medical rehabilitation services, falls short of its definition of ‘restoring someone to health or normal life to former condition’. The push, however, with PR and wider rehabilitation programmes is to encourage acceptance of a ‘new normal’, and the continuation of activity even at diminished capacity. While further work is needed to uncover what normal might look like for patients of varying socio-cultural backgrounds including in those communities where breathlessness may be lived as a ‘normal illness’,^13^ rehabilitation offers hope from connotations found elsewhere in understandings of chronic lung disease: where chronicity can be perceived as ‘there is nothing more that can be done’ or that ‘there is only going downhill from here’. In this sense, there are calls for goal-oriented forms of PR.^21^ However, the proposal for target-based PR is further complicated given that perceptions of breathlessness are subjective, and levels of dyspnoea-severity might increase even as personal goals are achieved and extended.

The perception of breathlessness then is a key tool from which PR is assessed.^22^ *Life of Breath* research is showing how life experiences, which can include anxiety or anticipation of an acute exacerbation, can shape how breathlessness is sensed through and with the body.^13^ For example, those living with breathlessness often pace their lives in anticipation of, and to limit threat of, an exacerbation. This affects the ways people engage with PR, through how they participate in sessions, or by choosing what events they would like to attend. For example, one individual chose to attend a support group, which was close to local shops, but not a community fitness group which was further away. The availability of parking or ease of public transport are also key issues. They were hesitant to attend PR without oxygen. This monitoring can be embodied as a constant mode of hyper vigilance of breathless symptoms, which could shape perception of the quality of breathlessness sensation and the subjective meaning of breathlessness.^23^

Outcome and performance measures, standardised within PR and wider respiratory care might also bear rethinking, processes which could benefit from being informed by a patient-led, medical humanities methodology.^24^ In this mode of inquiry, Havi Carel, co-PI on *Life of Breath*, uses philosophy to develop a toolkit to support patients in understanding their experiences of illness.^14^ Carel notes that illness can be seen to reframe structures of perception, and her toolkit uses an approach derived from the philosophical approach of phenomenology which enables reflection upon experiences of breathlessness, its impact and meaning. Her argument is that while patients’ understanding of their illness is often informed by clinical information and interactions, their actual life experience of their condition and the meanings that generates for them can be contradictory. Using this kind of deep reflection on their own experience, giving it validity alongside clinical knowledge, can be empowering for patients especially in contexts where management options are few. Further testing is underway with clinical collaborators to determine the most effective rollout of this toolkit, but it may offer an innovative and practical means to provide insight into patient perspectives and perceptions of breathlessness.

Carel’s more radical suggestion is that a shift is required in how chronic illness is ‘managed’ in a new context where more people are living with enduring conditions and cancer survivorship. This shift is one of ‘from cure to care’: from the notion that treatment is about an intervention intended to produce an end point ‘cure’ to one that is an ongoing learning process.^15^ An approach that takes into account the body’s capacity for adaptation means that management options might be more effectively seen as educational co-operation between clinician and patient, encouraging a life-long approach to change that enables chronically ill patients to find new ways of living and moving in their environments, achieving their goals and finding joy even in altered life circumstances.

### Stigma

Breath Lab respondents seemed to voice frustration with current clinical options by describing how the meaning of ‘rehabilitation’ can impact experiences in unhelpful and unintended ways. Many reported that that the term ‘rehabilitation’ can also carry the unfortunate and unwarranted stigma held by drug or alcohol rehabilitative services. This can influence experiences of shame (particularly considering steroidal treatments can mark the body in ways reminiscent to illegal drugs) or add to the hesitancy of PR uptake. Our work has corroborated other research that for many, shame is an intrinsic part of living with a lung condition and can be a barrier to PR, particularly where smoking has been a contributory factor to disease,^25–27^ or where invisible illness becomes unpleasantly perceptible through coughing, wheezing and the diminishment of capacity.^27,28^ The medical humanities can provide much needed insight into the experience of shame^29^ and breathlessness, noting that subjective and cultural factors shape the patient journey.^30^ For example, those with breathlessness have recalled finding comfort through humour in the confusion between rehabilitative treatments while others have felt uneasy and ashamed. In pacing their lives, some with breathlessness use ‘safe spaces’ to limit exposure to stigma, and when noting how those with breathlessness may also be anticipating an exacerbation or feelings of shame, medical humanities insights could help us understand more about how PR could become a safe space for pushing personal limits achieving health benefits. ‘Rehabilitation’ also assumes an aim that is directed at restoration of a previously functioning state. For many with chronic breathlessness, this must seem an impossible task that defeats them at the outset and induces feelings of failure. Rethinking the approach of PR to think of it as life-long learning might well be useful.

## Rethinking the label and/or offer of PR

Concerns of the term ‘pulmonary rehabilitation’ could be interpreted as implying that a revision is needed to encourage participation. There have certainly been efforts to make PR more easily understood with rebranded or overarching programmes, such as Newcastle’s ‘healthy lungs’ or Bristol’s ‘LEEP’ Lung Exercise and Education Programmes.^31^ This, however, still retains the inference that it is lung centred and could make the lungs healthier. *Life of Breath* research is also revealing that PR is often described in medicinal terms by both clinician and patient: the programme is ‘prescribed’, involves a ‘dose’ of exercise (in terms of each session or the 6–8 week course) and there is the notion that exercise is a one-off remedy. As one patient says, why would the name of PR need to be changed if it is seen as a medicine given that all other medicines have complicated names too. There certainly seems a tension between viewing PR as medicinal or limiting pathology versus approaching it as a key component of self-management that involves extended commitment; in practice one can be perceived as the sphere of professional clinical knowledge and the other appears to be aligned more closely with the daily lives of those living with a lung condition. In short, if PR is perceived as a course of treatment, seen to assist diseased lungs, this does not fully take into account additional longer-term health goals of the patient which should include ‘physical, mental and social well-being’^32^ – despite enhanced quality of life being the primary goal of the programme.

While it may appear useful to change the term ‘pulmonary rehabilitation’ to reflect the whole body, fitness approach it provides, more useful still would be to analyse those processes by which PR is received by patients, or those activities that are typically supported in a PR programme. If the lives of those living with breathlessness are taken as the primary concern, what amendments or alternatives to PR might be more effective at promoting patient health including, but not confined to, physical fitness? This question – or at least its latter emphasis – while underexplored is not novel,^33^ and studies are testing individualised online programmes,^34^ or incorporating cognitive behavioural therapy into the referral process.^35^ There is also emerging evidence that psychosocial and physical movement brings meaning to the lives of those with COPD, where individualised activity outside the confines of a secondary care setting may be more constructive and valued.^36^ Two emerging programmes which are less disease-focused involve singing and dance sessions tailored for those living with chronic breathlessness.

### Singing for breathing

Interest is developing in Singing for Lung Health (SLH) or ‘singing for breathing’ which involves patients with breathlessness taking part in singing groups facilitated by a trained instructor. The terminology once more points to the lungs, but the focus here is on the learning of breathing control and postural techniques that enable effective singing, where song is utilised as a tool to develop skills to assist with breathlessness.^37^ Furthermore, by emphasising singing for *breathing*, rather than for *breathlessness*, despite this being the underlying reason for SLH, the connotation found in much clinical and popular literature is avoided, namely where breathlessness is implicated as ‘an absence of breath’, reducing this complex process to its mechanistic conceptualisation.^16^ This point is important as singing can allow for patients who often experience their breath as negative and limiting, to reinterpret this association through the ‘positive achievement of song’.^38^

A recent consensus statement on the clinical benefits of SLH has picked up on some of the richness involved in the sensation and experience of breathlessness by stressing the potential psychological, social and physical improvements that can be gained from taking part in the programme.^38^ While research is required to fully conceive the physiological effects of taking part, physical benefits can include enhancing sputum clearance, breathing pattern modification and enhanced physical well-being. SLH can also provide a reduction in social isolation and levels of anxiety, which have been identified as major factors living with a lung condition,^39–42^ and continued participation might be encouraged by the inclusive social environment as well as performative aims of classes.^31^ Integration with PR is being considered,^38^ although the medical humanities perspective endorsed through the *Life of Breath* would consider that there is much needed scope to research the potential of SLH to be shaped to encourage participation from diverse communities, acknowledging that culturally meaningful connections improve PR uptake.^37^

### Dance movement

While aerobic dance therapy has been acknowledged as beneficial for those with lung disease, including as part of PR,^43^ it is still a relatively new approach to consider dance as a potential alternative to conventional exercise interventions within PR, or as a substitute or complementary programme.^44,45^ Reviews have already indicated that dance participation can assist young people and older adults improve their aerobic power, muscle endurance, flexibility and strength, balance, gait and agility, enhancing subjective well-being.^46–49^ Dance also promotes healthy ageing through facilitating social interaction, enjoyment and appreciation of aesthetics and mobility, which is encouraging for those living with chronic breathlessness.^46^ Dance as exercise appears to be culturally relevant, with previous successful interventions including Greek, Turkish, Korean, Cantonese and line dancing.^47^ Dance movement and SLH approaches thus both offer the potential for more engaged participation, particularly for those with lung disease who have fond memories of dancing or singing. Ethnographic research contributing to the *Life of Breath* project has already observed how various choreographies inform many of the warm-up and cool-down components of community exercise group sessions for those with breathlessness to warm reception. Yet dance also offers the unique opportunity to improve bodily awareness through movement and potentially to improve the accuracy of symptom perception.^50^ Initial studies have shown cognitive as well as psychological, physical and social improvements for those with dementia and Parkinson’s disease who have undertaken dance programmes.^50–53^ This is relevant given that certain lung conditions such as COPD have been proven to impact cognitive function,^54^ and that patients with cognitive impairment are less likely to complete a course of PR.^55^ Exercise has been shown to improve cognitive function in people with COPD.^56^ Cognitive impairment can also be concurrent with anxiety and negative perceptions of breathlessness,^57^ and thus dance could offer an effective intervention that allows for greater understanding of improving interoception, and perception of breathlessness through its holistic, whole body approach to fitness. *Life of Breath* is carrying out a pilot programme to examine the acceptability of dance as an adjunct to PR and to examine its effects on interoceptive awareness. This research, which integrates insights from humanities and sciences will further test these associations as well as the feasibility and value of a dance intervention that is culturally relevant and takes into consideration factors such as age, gender and symptom severity.

## Conclusion

This article has highlighted the value of a medical humanities approach in investigating issues surrounding patient engagement with PR services. This includes communication challenges in the translation of PR between patients and clinicians, and in those identified in diverse ‘clinical cultures’ within respiratory medicine which may have different approaches and aims. It offers practical recommendations for further research including a phenomenological toolkit and interdisciplinary investigation of SLH and particularly dance movement initiatives, which require a range of expertise to look into their cultural, linguistic and subjective relevance. To understand further what living well with breathlessness looks like and how to encourage this within communities, there is a need for collaborative research exploring the communication and meaning of healthcare strategies and experiences. This includes learning more about how breathlessness is lived day to day, what self-management and fitness means in various contexts, and how language, movement and experience are a central part of the patient journey. Encouraging holistic well-being must involve an open and integrated approach at the interface of the arts, humanities and medical practice.
